# Genomic sequencing and analyses of *Lymantria xylina *multiple nucleopolyhedrovirus

**DOI:** 10.1186/1471-2164-11-116

**Published:** 2010-02-18

**Authors:** Yu-Shin Nai, Chih-Yu Wu, Tai-Chuan Wang, Yun-Ru Chen, Wei-Hong Lau, Chu-Fang Lo, Meng-Feng Tsai, Chung-Hsiung Wang

**Affiliations:** 1 Department of Entomology, National Taiwan University, Taipei, Taiwan; 2 Department of Zoology, National Taiwan University, Taipei, Taiwan; 3 Department of Molecular Biotechnology, Dayeh University, No 112, Shanjiao Rd, Dacun, Changhua, Taiwan; 4 Department of Plant Protection, Faculty of Agriculture, Universiti Putra Malaysia, 43400 UPM Serdang, Selangor, Malaysia

## Abstract

**Background:**

Outbreaks of the casuarina moth, *Lymantria xylina *Swinehoe (Lepidoptera: Lymantriidae), which is a very important forest pest in Taiwan, have occurred every five to 10 years. This moth has expanded its range of host plants to include more than 65 species of broadleaf trees. LyxyMNPV (*L. xylina *multiple nucleopolyhedrovirus) is highly virulent to the casuarina moth and has been investigated as a possible biopesticide for controlling this moth. LdMNPV-like virus has also been isolated from *Lymantria xylin*a larvae but LyxyMNPV was more virulent than LdMNPV-like virus both in NTU-LY and IPLB-LD-652Y cell lines. To better understand LyxyMNPV, the nucleotide sequence of the LyxyMNPV DNA genome was determined and analysed.

**Results:**

The genome of LyxyMNPV consists of 156,344 bases, has a G+C content of 53.4% and contains 157 putative open reading frames (ORFs). The gene content and gene order of LyxyMNPV were similar to those of LdMNPV, with 151 ORFs identified as homologous to those reported in the LdMNPV genome. Two genes (Lyxy49 and Lyxy123) were homologous to other baculoviruses, and four unique LyxyMNPV ORFs (Lyxy11, Lyxy19, Lyxy130 and Lyxy131) were identified in the LyxyMNPV genome, including a *gag-like *gene that was not reported in baculoviruses. LdMNPV contains 23 ORFs that are absent in LyxyMNPV. Readily identifiable homologues of the gene *host range factor-1 *(*hrf-1*), which appears to be involved in the susceptibility of *L. dispar *to NPV infection, were not present in LyxyMNPV. Additionally, two putative *odv-e27 *homologues were identified in LyxyMNPV. The LyxyMNPV genome encoded 14 *bro *genes compared with 16 in LdMNPV, which occupied more than 8% of the LyxyMNPV genome. Thirteen homologous regions (*hr*s) were identified containing 48 repeated sequences composed of 30-bp imperfect palindromes. However, they differed in the relative positions, number of repeats and orientation in the genome compared to LdMNPV.

**Conclusion:**

The gene parity plot analysis, percent identity of the gene homologues and a phylogenetic analysis suggested that LyxyMNPV is a Group II NPV that is most closely related to LdMNPV but with a highly distinct genomic organisation.

## Background

Baculoviruses (family *Baculoviridae*) consist of rod-shaped, arthropod-specific viruses with double-stranded, circular DNA genomes ranging from 80 to 180 kb and enveloped nucleocapsids [1]. The occluded form of the virus is embedded in proteinaceous occlusion bodies (OBs) [1]. *Baculoviridae *is subdivided into four genera, Alphabaculovirus (lepidopteran-specific nucleopolyhedrovirus, NPV), Betabaculovirus (lepidopteran-specific granulovirus), Gammabaculovirus (hymenopteran-specific NPV) and Deltabaculovirus (dipteran-specific NPV) [2]. The lepidopteran-specific NPVs are further classed into two groups, I and II, based on the phylogenetic analysis of their polyhedrin (*polh*) genes [3,4]. Most of the NPVs with sequenced genomes are lepidopteran specific. However, the genomes of *Culex nigripalpus *NPV, which infects dipteran, and three Neodiprion NPVs which have been isolated from hymenopteran insect species, have also been sequenced [5-8]. To date, more than fifty baculovirus isolates have been sequenced (NCBI, GenBank). Baculoviruses are used worldwide as protein expression vectors, biotechnological tools and biological control agents of agricultural and forest pests.

The casuarina moth, *Lymantria xylina *Swinehoe (Lepidoptera: Lymantriidae), is an herbivore that feeds on casuarina (*Casuarina equisetifolia*), guava (*Psidium guajava *L.), longan (*Euphoria longana *Lam.), lychee (*Litchi chinensis *Sonn.), acacia (*Acacia confusa*) forests and more than 60 other species of host plants [9,10]. It is native to Taiwan, Japan, India, and the eastern coast of mainland China [11]. In the last 30 years in Taiwan, many forests have been converted to agricultural land. The moth has expanded into these newly established agricultural areas and simultaneously expanded its host plant range [12]. *L. dispar *is a closely related species to *L. xylina *[13]. The *L. dispar *MNPV (LdMNPV) isolated from its natural host, *L. dispar*, had been characterised and sequenced in 1999 [14] and used in integrated pest management (I.P.M.) programs to control this pest for many years in America [15].

NPV epizootics occur in populations of *L. xylina *each year from spring to early summer in Taiwan and mainland China, and the key pathogen was found to be *L*. *xylina *multiple nucleopolyhedrovirus (LyxyMNPV) [16]. Following the establishment of an *in vitro *propagation system for LyxyMNPV in the cell lines IPLB-LD652Y (LD) and NTU-LY (LY) [17,18], LyxyMNPV and host-virus interaction studies were promoted. In previous tissue culture infectivity studies of three lymantriid NPVs (LyxyMNPV, LdMNPV, and *Orgyia pseudotsugata *MNPV), only one cell line, IPLB-LD-652Y, was able to support infection and replication of all three NPVs [17,19,20]. Additionally, *Perina nuda *NPV (PenuNPV), which was isolated from *P. nuda*, could also infect both LY and LD cells and *L. xylina *larvae in our laboratory [18]. Of these Lymantriidae-derived NPV species, LyxyMNPV/LdMNPV and PenuNPV/OpMNPV were thought to be closely related [17,21]. However, molecular evidence has supported them as distinct species [12,16]. Therefore, the precise relationship between LyxyMNPV and LdMNPV needs to be further clarified. In our previous study, LyxyMNPV and another less prevalent NPV species were shown to coexsist in the infected larvae in the fields of Taiwan [12]. The less prevalent NPV was isolated and characterised as LdMNPV-like virus [12].

In this study, we reported the complete genomic sequence of LyxyMNPV, which is the most prevalent virus strain in the infected *L. xylina *larvae and described the whole genomic sequence, gene structure and performed a phylogenetic analysis. The genome sequences were compared to the previously published LdMNPV [14] and group I NPV type species, *Autographa californica *MNPV [22]. Because OpMNPV [23] is another Lymantriidae-derived NPV species that can infect LD cells and *Maruca vitrata *MNPV (MaviMNPV) [24] can also infect LD cells at low level, these two group I NPVs were also compared to LyxyMNPV.

## Results and Discussion

### General characteristics of the LyxyMNPV genome

**Figure 1:**
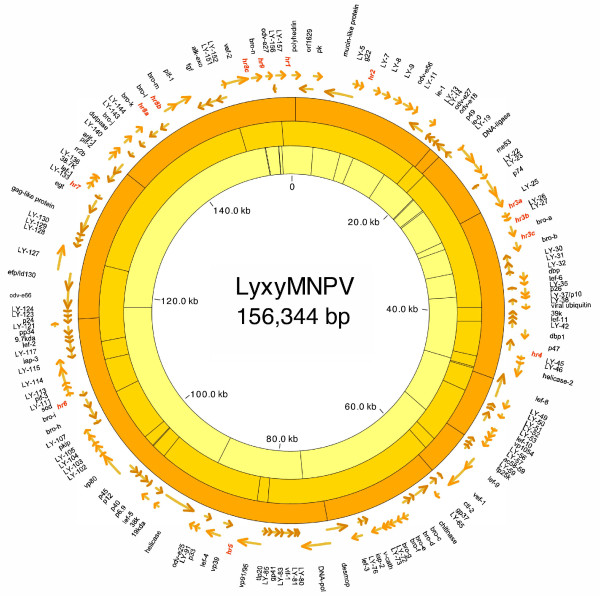
**Circular map of the LyxyMNPV genome**. The inner circles indicate the positions of cleavage sites for the following enzymes: outer circle, *Bam*HI; middle circle, *Eco*RI; inner circle, *Eco*RV. LyxyMNPV ORFs are indicated outside these circles. Arrows indicate the direction of transcription. The locations of *hrs *are also shown. A scale in kb is provided in the centre of the figure.

The LyxyMNPV genome size is 156, 344 bp [GenBank: GQ202541] and has a G+C content of 53.4% (see Additional file 1). ORFs were predicted according to the initial criteria for further study. However, three ORFs that had large overlap found in LyxyMNPV were also selected for further study, namely Lyxy37, Lyxy72 and Lyxy139 (*arif-1*). Lyxy37 (281 aa) overlaps with Lyxy36 (*p26*) by 192 aa in the same direction, but not in the same frame. A P10 homologue (77 aa) was found located in the C-terminal portion of Lyxy37 (from 205 aa to 281 aa), designated as Lyxy37'. Lyxy72 (homologous to Ld76) is 380 aa long and overlaps with Lyxy71 (*bro-g*) and Lyxy73 by 182 aa and 119 aa, respectively. Lyxy139 (*arif-1*), which is 272 aa long, overlaps with Lyxy138 (*pif-2*) and Lyxy140 by 182 aa and 73 aa, respectively. Therefore, a total of 157 ORFs were identified for further analysis (Fig. 1, Additional file 2), and nucleotides in the LyxyMNPV genome were numbered sequentially, beginning with the A (designated position 1) of the *polyhedrin *start codon (ATG). The directions of the transcripts are indicated by arrows. The ratio of the ORF orientations was almost equal to 1:1 [clockwise (79/157): anticlockwise (78/157)] for those oriented clockwise with respect to the orientation of the *polh *gene [25] (ORF1). Most of the 157 LyxyMNPV ORFs have an assigned function or homologues in other baculoviruses. As shown in Table 1, four unique ORFs were found in the LyxyMNPV genome. There are 30 conserved genes in all baculovirus genomes, including the dipteran and hymenopteran baculoviruses [6,26,27] and all of these genes were found in the LyxyMNPV genome. A total of 14 baculovirus-repeated ORFs (*bro *genes, *ly-bro-a *to *ly-bro-n*) were also sequenced. Beside these 157 predicted ORFs, other internal spaces were made up of intergenic spaces and *homologous region*s (*hr*s). The LyxyMNPV genome has 13 *hrs *(*hr1, 2, 3a, 3b, 3c, 4, 5, 6, 7, 8a, 8b, 8c *and *hr9*) (Fig. 1 and Additional file 2), and the orientations of the *hrs *were related to those of LdMNPV (Additional file 2).

**Table 1:** Six genes of LyxyMNPV with no homologue in LdMNPV genome.

LyxyMNPV			
ORF*	**Length (aa)**	**Homologue**	**Identity (%)**

11	58		
19	55		
49	305	*Clbi31*	15
123	105	*Agse52*	35
130	83		
131	689	*Bombyx mori/gag-like*	18

* LyxyMNPV unique genes are underlined.

### Promoter analysis

Promoter motifs present upstream of the 157 putative ORFs were screened. 14 LyxyMNPV ORFs were shown to possess consensus early gene promoter motif (Additional file 2). Three of these ORFs, Lyxy7, Lyxy10 (*odv-e56*) and Lyxy126 (*efp/ld130*), were also shown to possess consensus late gene promoter motif, which may allow transcription of these genes during both the early and late stages of infection. This has also been reported for Ac128 (*gp64*) and Ac147 (*ie-1*) [28,29]. A total of 73 LyxyMNPV ORFs were shown to possess consensus late gene promoter motif. Additionally, the upstream 210 bp of 50 LyxyMNPV ORFs had an enhancer-like element consisting of a CGTGC motif (Additional file 2). A total of 48 LyxyMNPV ORFs did not possess consensus late or early promoter sequences. It seems likely that additional, as-yet-unidentified, promoter sequences might exist within the LyxyMNPV genome, as in other baculoviruses [28,29]. Unlike most baculoviruses, the *p74 *of LyxyMNPV lacks a late promoter [14].

### Comparison of LyxyMNPV ORFs to other baculoviruses

LyxyMNPV shares 109 ORFs with AcMNPV, 100 ORFs with OpMNPV, 93 ORFs with MaviMNPV, 151 with LdMNPV, 107 with SeMNPV and 76 with CpGV (*Cydia pomonella *GV). The average amino acid sequence identity between LyxyMNPV and AcMNPV [22], OpMNPV [23], MaviMNPV [24], LdMNPV [14], SeMNPV [30] and CpGV [31,30] were 32.68%, 33.19%, 33.28%, 74.71%, 37.3% and 25.63%, respectively. Four ORFs (Lyxy11, Lyxy19, Lyxy130 and Lyxy131) were identified unique to LyxyMNPV (Additional file 2). Three pairs of adjacent AcMNPV ORFs (Ac58/Ac59, Ac106/Ac107, and Ac112/Ac113) were fused together into single ORFs (Lyxy58, Lyxy115 and Lyxy105, respectively) in LyxyMNPV. As reported for *Rachiplusia ou *MNPV-R1, re-sequencing of these regions in AcMNPV-C6 had found that the ORF pairs occurred as a single ORF in the stock of AcMNPV-C6 [32]. The homologues of these ORFs were also found in other baculovirus genomes in which they are fused into a single ORF. Three adjacent LdMNPV ORFs (Ld133/Ld134/Ld135) that were in the same orientation were also fused into a single ORF (Lyxy122) in LyxyMNPV.

### GeneParityPlot analysis

**Figure 2:**
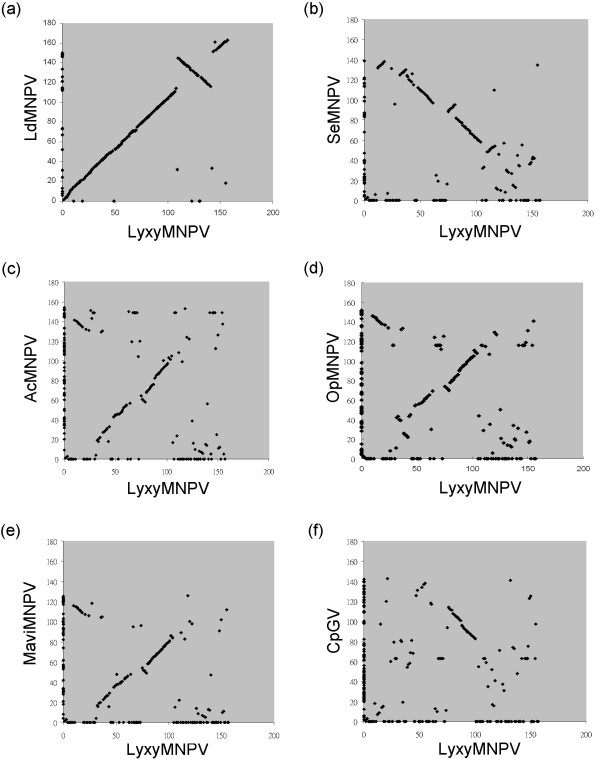
**Gene parity plots comparing ORF content and order of LyxyMNPV with (a) LdMNPV, (b) SeMNPV, (c) AcMNPV, (d) OpMNPV, (e) MaviMNPV and (f) CpGV**. ORFs present in only one of the compared genomes appear on the axis corresponding to the virus in which they are present.

Comparisons of the gene arrangement of the selected ORFs are shown in Fig. 2. The gene arrangement of the LyxyMNPV genome was highly colinear to that of LdMNPV. However, a large fragment flanked by Lyxy109 (*bro-i*) and Lyxy142 (*bro-j*) in the LyxyMNPV genome showed an inversion compared to LdMNPV (Fig. 2a). The gene arrangement of the LyxyMNPV genome showed lower colinearity to AcMNPV, OpMNPV, MaviMNPV and SeMNPV. In contrast, the parity analysis of the LyxyMNPV and CpGV ORFs displayed a much more dispersed pattern.

### LyxyMNPV structural genes

Compared to the 25 baculovirus structural genes listed by Hayakawa et al. (1999), Herniou et al., (2003) and Jehle et al. (2006)[2,26,33], only two genes were identified to be absent in the LyxyMNPV genome. These genes included the protein tyrosine phosphatase gene (*ptp*) and group I NPV-specific *gp64 *(see Additional file 3), which is the major envelope fusion protein gene of AcMNPV [22], *Bombyx mori *MNPV (BmNPV) [34], OpMNPV [23] and *Epiphyas postvittana *NPV (EppoNPV) [35]. The GP64 protein appears to be unique to group I NPVs [36,37]. In LdMNPV, the F protein, an envelope fusion protein functionally analogous to the GP64 protein, is a product of the *Ld130 *gene. LyxyMNPV encodes an F protein homologue, Lyxy126, which shows 96% aa identity to *Ld130*. *Ld130 *homologues are present in all lepidopteran and dipteran baculoviruses that have been completely sequenced, including those that contain *gp64*. The role of the *Ld130 *homologue in the latter species is unclear, but it is likely that they have lost their envelope fusion properties [37,38]. Of these structural proteins, the polyhedrin protein was the most conserved (100% identity). In contrast, the products of genes *pif-2*, *orf1629 *and v*p80 *showed the lowest levels (75%, 71% and 69%, respectively) of sequence conservation between LyxyMNPV and LdMNPV (Additional file 2). A special characteristic of LyxyMNPV is that Lyxy15 and Lyxy155 are homologous to *odv-e27*. Lyxy15 is flanked by homologues of Ld17 and Ld19, while Lyxy155 is close to LyxyMNPV *hr9 *and located between Lyxy154 and Lyxy156 which are homologues of Ld161 and Ld162, respectively. The identity between Lyxy15 and its LdMNPV homologue (Ld18) is up to 84%. However, the identity between the two LyxyMNPV *odv-e27 *genes is quite low (18%) which is not significantly higher than that to the AcMNPV, MaviMNPV or SeMNPV even AnpeNPV homologues (Additional file 2). The region of Lyxy155 has probably undergone extensive rearrangement, because Ld18 homologue gene of other different baculoviruses is located in the similar site of Lyxy15. Therefore, it is possible that Lyxy155 was acquired from other NPV species (AcMNPV, MaviMNPV, SeMNPV or AnpeNPV). In AcMNPV, the ODV-E27 protein was incorporated into both the envelope and capsid of the occluded virus [39]. However, only *odv-e27 *(Lyxy15) of LyxyMNPV has a late promoter motif. Therefore, it is possible that only one *odv-e27 *is transcribed in LyxyMNPV, and that it serves as a structural protein of the envelope and capsid of an occluded virus. It is notable that *p10 *(designated as Lyxy37') of LyxyMNPV has a large N-terminal extension. The identity between Lyxy37' and its LdMNPV homologue (Ld41) is up to 87%, which is much higher than that of Lyxy37 and Ld41 (23%). Lyxy37 has early and late promoter motifs, but Lyxy37' has only one late promoter motif. There may be two forms of *p10 *found in the early and late stages of LyxyMNPV-infected larvae or cells. The average identity of these structural proteins was generally 86.5% between LyxyMNPV and LdMNPV (Additional file 2), suggesting that the structure of the viruses may be similar.

### Transcription-specific genes

The genes involved in baculovirus late gene transcription that are present on other baculovirus genomes are also present in the LyxyMNPV genome, with no exceptions, including *lef 4-6*, *8-11*, *39K*, *p47 *and *vlf-1 *[40]. These genes are found in LyxyMNPV as Lyxy89, Lyxy96, Lyxy34, Lyxy48, Lyxy61, Lyxy54, Lyxy41, Lyxy40, Lyxy44 and Lyxy82, respectively. Most of these genes were 80%-96% identical in amino acid sequences to those of LdMNPV. However, we noted that the amino acid identity of LdMNPV *lef-6 *and LyxyMNPV *lef-6 *aa was only 64%.

### DNA replication and repair genes

A total of 19 *lef *genes in AcMNPV have been implicated in DNA replication and transcription [41]. Baculovirus early genes are transcribed by the host cell RNA polymerase II, but this is often transactivated by genes such as *ie-0*, *ie-1*, *ie-2 *and *pe38 *[42]. Of these genes, *ie-0 *and *ie-1 *are present in LyxyMNPV. Six baculovirus genes have previously been reported as essential DNA replication factors for baculoviruses, including *lef-1*, *lef-2*, *lef-3*, *DNApol*, *helicase *and *ie-1 *[43], which are all present in LyxyMNPV. These genes are conserved with respect to those of LdMNPV (81%-91%), with the exception of *lef-3 *(41%). Homologues of a single-stranded DNA-binding protein (*dbp*) [44] and an immediate-early gene, *me53*, both of which have been implicated in DNA replication, were also found in LyxyMNPV. Additionally, similar to LdMNPV, LyxyMNPV also has two *dbp *(Lyxy33 and Lyxy43). The amino acid sequence identities of the two DBP to those of LdMNPV were 75% and 80% for Lyxy33 and Lyxy43, respectively. However, the amino acid identity was very low between the two (19%). Genes for enzymatic functions in nucleotide metabolism, such as the large (*rr1*) and small (*rr2*) subunits of ribonucleotide reductase and deoxyuridyltriphosphate (*dutpase*), which are found in several baculoviruses, were also present in LyxyMNPV, with the exception of *rr1*. Three genes that encode ribonucleotide reductase were found in LdMNPV. These include a homologue of the large subunit and two homologues of the small subunit of ribonucleotide reductase copies [14]. The *rr2 *of LyxyMNPV (Lyxy137) was more similar to Ld120 (*rr2b*, 86%) than to Ld147 (*rr2a*, 16%) homologues. These enzymes are involved in nucleotide metabolism and catalyse the reduction of host cell rNTPs to dNTPs [45]. In addition, the homologues of non-essential DNA replication stimulatory genes, *ie-2*, *lef-7 *and *pe38 *[46], which were unique in group I NPV, were not present in LyxyMNPV.

### Genes with auxiliary functions

Auxiliary genes are not essential for viral replication, but provide a selective advantage to increase virus production/survival either at the cellular level or at the level of the organism [47]. A total of thirteen auxiliary genes have homologues in LyxyMNPV, including *proteinkinase-1 *(*pk-1*), *p10*, *ubiquitin *(*ubi*), *conotoxinlike peptide-2 *(*ctl-2*), *chitinase*, cathepsin L-like proteinase (*v-cath*), *superoxide dismutases *(*sod*), *viral enhancing factor-1*, (*vef-1*), *vef-2*, *ecdysteroid UDP glucosyltransferase *(*egt*), *actin rearrangement-inducing factor-1 *(*arif-1*), *viral fibroblast growth factor *(*vfgf*) and *alkaline exonuclease *(*alk-exo*). Almost all of these auxiliary genes in LyxyMNPV were 81%-95% identical in amino acid sequence to those of LdMNPV, except *arif-1 *and *ctl-2*. LyxyMNPV *arif-1 *was 64% identical to that of LdMNPV and its *ctl-2 *was 66% and 40% identical to OpMNPV and LdMNPV, respectively.

Conotoxins are small disulfide-rich ion channel antagonists isolated from the gastropod genus *Conus *[48]. A single *ctl *gene is present in AcMNPV (Ac3). In contrast, the LdMNPV and OpMNPV genomes encode two *ctl *genes, called *ctl*-1 and *ctl*-2. However, there is only one *ctl *gene (*ctl-2*) found in LyxyMNPV, which has low amino acid identity to LdMNPV. This is because LdMNPV *ctl*-2 has a longer amino acid size (92 aa) than the other baculovirus *ctl*s. The biological function of CTL remains unclear, as no difference in mortality, motility, or weight gain was observed when neonate or late instar *Spodoptera frugiperda *larvae were infected with the AcMNPV *ctl-1 *deletion mutant compared to infection with wild-type virus [49].

The large VEF protein is metalloprotease that appears to degrade mucin and is thought to facilitate the approach of baculoviral virions to the surface of gut cells by disrupting the peritrophic membrane [50]. This protein forms ~5% of the mass of OBs of the *Trichoplusia *GV [51]. The *vef *gene does not exist in the AcMNPV [22] or OpMNPV [23] genomes, but LdMNPV has two *vef *genes, *vef-1 *and *vef-2*. Previous studies have revealed that both LdMNPV enhancins contributed to viral infectivity [52,53]. Two copies of *vef *(*vef-1 *and *vef-2*) have been identified in the LyxyMNPV genome (Lyxy62 and Lyxy153). The two gene products show low amino acid identity to each other (29%). Auxiliary genes that are present in other lepidopteran NPVs but are not in LyxyMNPV include *pcna*, *pk-2*, *ptp-1 *and *ctl-1*.

### Anti-apoptosis genes

Apoptosis or cell death can be a mechanism to defend against the establishment of infections and is usually triggered in the early period of virus infection. Baculoviruses possess two families of genes that suppress apoptosis: the *p35*/*p49 *family and the inhibitor of apoptosis (IAP) family. It has been shown that *p35 *blocks diverse pathways of apoptosis and plays a role in blocking AcMNPV-induced apoptosis in Sf21 cell [54]. This gene has been reported in a number of baculoviruses, such as AcMNPV, BmNPV, RoMNPV, *S. litura *MNPV (SpltMNPV) and MaviMNPV [22,24,32,34,55].

The *iap *family of genes have been found in all family members of the Baculoviridae sequenced to date. IAP homologues generally contain two baculovirus IAP repeats (BIR), which are associated with binding to apoptosis-inducing proteins, and a C-terminal zinc finger-like (RING) Cys/His motif [56]. These features have enabled the *iap *genes to be divided into five groups from *iap-1 *to *iap-5 *[31]. Apoptotic inhibition has been recovered in AcMNPV *p35 *deletion mutants with a variety of baculovirus *iap *homologues [57]. In the LyxyMNPV genome, two *iap*s were observed, *iap-2 *(Lyxy75) and *iap-3 *(Lyxy116). Both *iap*s only have one BIR domain and one RING domain in the predicted amino acid sequences.

### Baculovirus repeated ORFs (*bro *genes)

A striking feature of most lepidopteran and dipteran NPVs sequenced to date and in some of the GVs is the presence of one to 16 copies of *bro *genes. Typically *bro *genes are highly conserved, repetitive and widely distributed amongst insect DNA viruses [58]. The function of these genes is unclear, but they have been shown to bind to DNA [59]. These genes have also been found to be associated with the regions of viral genome rearrangement [60]. LyxyMNPV contains 14 *bro *genes, which have been named *bro-a *to *bro-n *based on their order in the genome (Fig. 1; Additional file 2). Most BROs contain a core sequence of 41 aa at the N-terminal half and several different domains throughout the sequence. The *bro *gene family has been divided into four groups based on the similarity of those domains [14]. Six of the LyxyMNPV bro genes, including *ly-bro-b *(Lyxy29), *-h *(Lyxy108), *-j *(Lyxy142), *-k *(Lyxy145), *-l *(Lyxy146) and *-n *(Lyxy154) which are homologues of *ld-bro-b*, *-j*, *-b*, *-p*, *-n *and *-p*, respectively, belong to group I, while three *bro*s [*ly-bro-a *(Lyxy28), *-i *(Lyxy109) and *-m *(Lyxy147) which are homologues of *ld-bro-a*, *-a *and *-o*, respectively] belong to group II. Four of them, including l*y-bro-c *(Lyxy67), *-d *(Lyxy68), *-e *(Lyxy69), and *-f *(Lyxy70) which are homologues of *ld-bro-c*, -c, *-c *and *-d*, respectively, belong to group III. Only one *bro *[*ly-bro-g *(Lyxy71) which is homologues of *ld-bro-g*] belongs to group IV. The homologues of *ld-bro-f*, *-h*, *-i*, *-k*, *-l *and *-m *are not presented in LyxyMNPV genome. However, in LyxyMNPV, all group III *bro *genes encode small fragments of truncated protein (154 aa, 330 aa, 196 aa and 249 aa). *Ly-bro-c*, *ly-bro-d *and *ly-bro-e *are homologous to different regions of *ld-bro-c*, but *ly-bro-f *is homologous to the N-terminal portion of *ld-bro-d*.

### Homologous regions (*hrs*)

A novel feature of many baculovirus genomes is the presence of homologous regions (*hrs*) located throughout the genome [61]. A single *hr *comprises a palindrome that is usually flanked by direct repeats and is closely related to counterparts located elsewhere in the genome. According to the transient replication assays, *hrs *may play a role in the replication origins of NPVs and GVs [62] and function as enhancers of RNA polymerase II-mediated transcription of baculovirus early promoters in NPVs [63]. It has been suggested that *hrs *can probably substitute for each other. However, it was recently shown that no single homologous repeat region is essential for DNA replication of AcMNPV [64].

**Figure 3:**
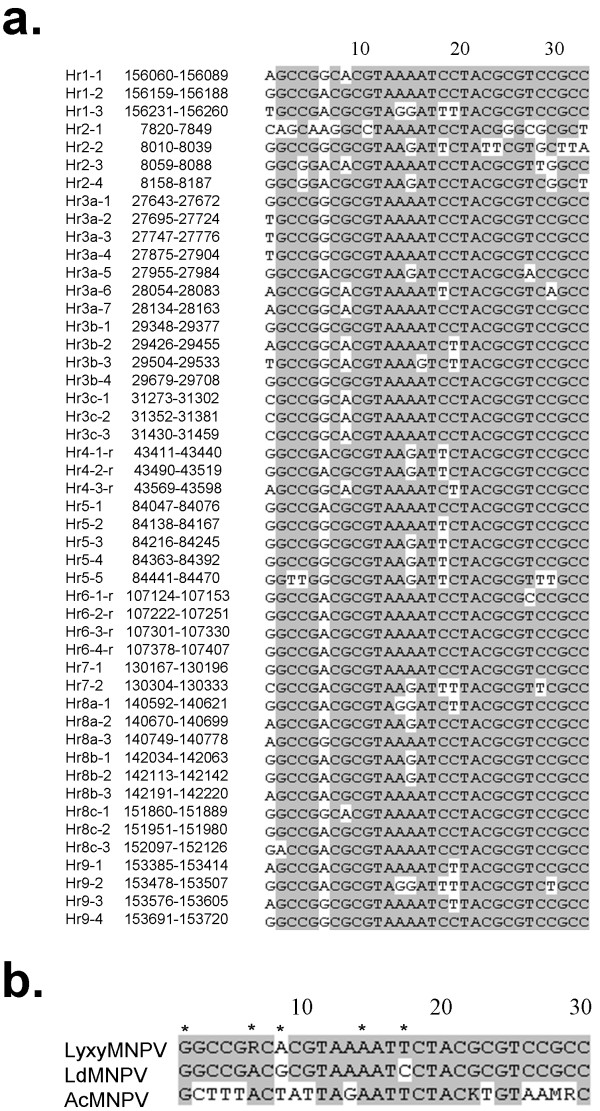
**Comparison of LyxyMNPV *hr *palindromes with the *hr *palindrome consensus sequence from other baculoviruses**. (a) Alignment of 48 palindrome repeat sequences in the LyxyMNPV genome. Black letters on grey: >80% conserved; "r": reversed sequence; (b) Alignment of the consensus *hr *palindrome from LyxyMNPV, LdMNPV[14] and AcMNPV [25]. The asterisks above the sequence indicate the variations of the palindrome. R = A or G, K = G or T, M = C or A.

The LyxyMNPV genome contains thirteen homologous repeat regions (*hr1*, *hr2*, *hr3a*, *hr*3*b*, *hr*3*c*, *hr4*, *hr5*, *hr6*, *hr7*, *hr8a*, *hr*8b, *hr8c *and *hr9*) that include two to seven palindrome repeats for a total of 48 repeats (Fig. 3a) and account for 7.3% of the genome. Of these *hrs*, *hr4 *and *hr6 *are in the reverse direction compared to other *hrs *in the LyxyMNPV genome (Fig. 3a). Similar to the LdMNPV palindrome sequence [14], the LyxyMNPV *hr *palindrome consensus GGCCGRCACGTAAAATTCTACGCGTCCGCC shows 24/30 palindrome matches (Fig. 3b) and the palindromic consensus sequence includes five highly variable positions (Fig. 3b). All nucleotides in the palindrome are >91.5% conserved, except for the eighteenth nucleotide (81.3% conserved), and with the variable nucleotides being 50%-77% conserved. In addition, the AcMNPV consensus *hr *palindrome has 50% sequence identity with LyxyMNPV consensus *hr *sequence (Fig. 3b).

**Figure 4:**
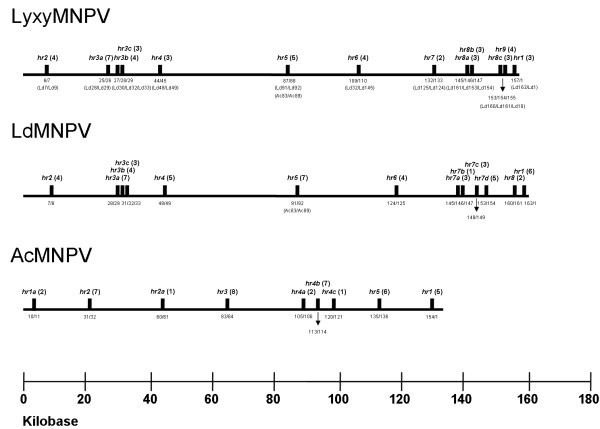
**Comparison of the genomic context of the *hrs *and *hr *locations relative to homologous ORFs between LyxyMNPV, LdMNPV **[14]** and AcMNPV **[25]** in the linearised genomes**. Black bars indicate the locations of *hrs *in the linearised genomes. ORFs flanking the *hrs *are indicated below the line. AcMNPV *hr2a *is shown as in Possee & Rohrmann [84]. For consistency, all linearised genomes start with *polh*, but *hrs *and ORF numbers remain as in the original papers. The numbers in brackets indicate the number of complete repeats in the *hr *palindromes. A scale in kb is provided in the bottom of the figure

**Figure 5:**
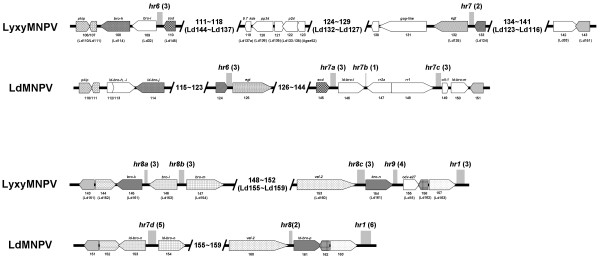
**Detailed *hr *locations and gene organisation in the LyxyMNPV genomic fragment ranging from Lyxy106 to the *hr1 *region relative to those of the LdMNPV genome**. The number and transcriptional direction of each ORF are labelled by arrows indicating the direction of transcription. Each pair of LyxyMNPV and LdMNPV homologue genes is labelled in a different pattern. ORF numbers are shown below the arrows. The names of the putative genes are shown on the arrows. Grey rectangles indicate the locations and relative lengths of the *hr*s.

The genomic positions of LyxyMNPV regions *hr1 *- *hr5 *are conserved with the genomic positions of LdMNPV *hr1 *- *hr5 *[14]. LyxyMNPV *hr1*, *hr3a*, *hr3c*, *hr4 *and *hr5 *are flanked by the same ORFs as those in the LdMNPV genome (Fig. 4) and LyxyMNPV *hr7*, *hr8b *and *hr8c *are flanked by the same ORFs as LdMNPV *hr6*, *hr7d *and *hr8*, respectively (Fig. 5). Major insertions and deletions were found near the LyxyMNPV *hr2 *and *hr3b*. Therefore, LyxyMNPV *hr2 *is flanked by Lyxy6 (Ld7) and Lyxy7 (Ld9) and *hr3b *is flanked by Lyxy27 (Ld30) and Lyxy28 (Ld32) (*ld-bro-a*) (Fig. 4). Conversely, LdMNPV *hr7a*, *hr7b *and *hr7c *are not present in the corresponding locations in the LyxyMNPV (Fig. 5). As reported in other comparisons of closely related viruses, ORF content tends to differ markedly between LdMNPV and LyxyMNPV around the *hr6*, *hr8a *and *hr9*. LyxyMNPV *hr6 *is located between Lyxy109 (Ld32) and Lyxy110 (Ld145), *hr8a *is located between Lyxy145 (Ld161) and Lyxy146 (Ld153) and *hr9 *is located between Lyxy154 (Ld161) and Lyxy155 (Ld18) (Fig. 5). It is notable that in the region from Lyxy110 to Lyxy142, between *hr6 *and *hr7 *showed a reverse gene arrangement compared to those of LdMNPV (Fig. 2a; Fig. 5).

The positions of at least some *hr*s appear to be conserved relative to specific baculovirus genes. In particular, it was found that an *hr *is conserved immediately downstream of Ac83 and the homologous genes in BmNPV [34], OpMNPV [23] and LdMNPV [14]. LyxyMNPV *hr5 *is located immediately downstream of Lyxy87 (*vp91/p95*), the LyxyMNPV homologue of Ac83 (Fig. 4). In addition, similar to LdMNPV, because no homologue of the AcMNPV ORFs (Ac84-Ac88) are present in the LyxyMNPV genome, Lyxy88 is located immediately downstream of this *hr*, which is the homologue of AcMNPV *vp39 *(Ac89). This indicates that the position of LyxyMNPV *hr*5 is conserved with regard to both the upstream and downstream genes. The ORF organisation downstream of this *hr *in OpMNPV, BmNPV [34] and LdMNPV is similar to LyxyMNPV. Homologues of Ac84-Ac86 are missing in BmNPV, while Ac84 and Ac86 are missing in OpMNPV. Given that *hr*s share higher similarity within a virus strain than any *hr*s between species, this evidence further indicates that hrs play a fundamental role in viral life cycle and replication process appears to be tightly linked to functional conservation.

### LyxyMNPV duplicate genes

Two pairs of genes, Lyxy45/Lyxy46 and Lyxy50/Lyxy51, were identified as duplicated homologues of Ld49 and Ld53 in the LyxyMNPV genome. All of these duplicate genes show low identities to each other (20% and 6%). However, Ly45/Ly46 has higher identity (38%) from amino acid positions 51 to the 105, while Lyxy50/Lyxy51 has higher identy (17%) from amino acid 60 to the 126. Of these duplicate genes, Lyxy45 and Lyxy50 have low identities to Ld49 (17%) and Ld53 (45%), respectively but Lyxy50 with the C-terminal portion consisting of 81 amino acids has over 75% identity to Ld53. However, both Lyxy46 and Lyxy51 are 13% identical to the homologues of LdMNPV.

### Unique LyxyMNPV ORFs

Four genes are unique in the LyxyMNPV genome, including Lyxy11, Lyxy19, Lyxy130 and Lyxy131 (Additional file 2). Most of these ORFs are small in size (55-83 aa), with the exception of Lyxy131 (689 aa). Only Lyxy130 contain a recognisable promoter. Lyxy130 possesses a late gene promoter motif and an enhancer-like element. Those ORFs with no recognisable promoter may not be transcribed, but Lyxy130 may be transcribed during the early or late stages of infection and could be contributing factor in host range expansion and some pathology of LyxyMNPV. Both Lyxy11 and Lyxy19 have no baculovirus homologue and no significant BLAST database hit. Lyxy130 and Lyxy131 however have at least one significant BLAST database hit. Lyxy130 had a 32% identity match to 20 aa of a hypothetical protein [GenBank: XP_567060] of the fungal species *Cryptococcus neoformans *(the e-value is 25). Interestingly, Lyxy131 had a 31% identity match to 62 aa of *Drosophila melanogaster *GAG protein [GenBank: AAT12844] (the e-value is 5e-25), which shows some homology, albeit low (10, 18 and 14%, respectively) to full length of *D. melanogaster *and *B. mori *[GenBank: BAB21762], and it has also been identified in the highly repetitive elements (LDT1) of the gypsy moth (*L. dispar*) [GenBank: AAC72920] [65]. In retroviruses, the gag gene encodes structural proteins and will be primarily translated as Gag precursor that acts to generate structural proteins of the mature infectious virus. All retroviruses have at least three mature Gag proteins that are generically referred to as matrix (MA) protein, capsid (CA) protein, and nucleocapsid (NC) protein [66]. However, as mentioned above, the *gag *gene of LyxyMNPV has no recognizable promoter so it may not be transcribed and therefore may not contribute to the encapsidation of LyxyMNPV.

### Phylogenetic analysis of LyxyMNPV

**Figure 6:**
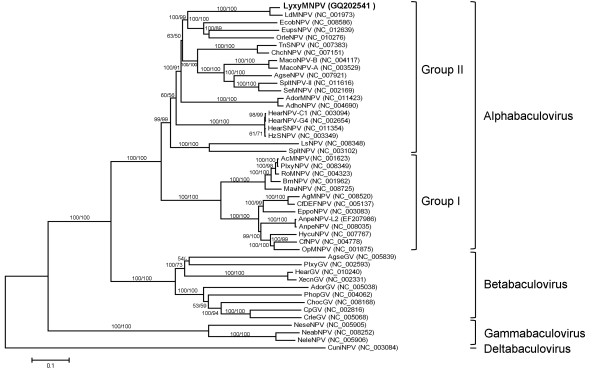
**Phylogenetic analysis of concatenated amino acid sequence alignments, showing bootstrap values >50% for NJ and MP trees at each node (NJ/MP)**. The location of LyxyMNPV is printed in bold.

The neighbour-joining (NJ) and maximum parsimony (MP) trees generated similar results, but the NJ tree revealed higher bootstrap values. The results reflect the current systematic assignment of the viruses. As shown in Fig. 6, the family *Baculoviridae *consists of five major clades: the NPVs infecting Lepidoptera (including group I and group II), the GVs, the Hymenopteran-specific NPVs and CuniNPV. Two subclades within the lepidopteran NPV group II resemble the LdMNPV and AdhoNPV lineages as reported by Oliveira et al. [67]. The result indicated that LyxyMNPV and LdMNPV are grouped together. These results correspond to our previous studies [12,16] and indicate that LyxyMNPV is a baculovirus distinct from LdMNPV but the two are closely related based on the pairwise distances of the nucleotide sequences of *polh*, *lef-8 *and *lef-9 *[12].

### Comparison of LyxyMNPV to LdMNPV

The most significant difference between LyxyMNPV and LdMNPV is a large genomic fragment (29.3 kb in length) of LyxyMNPV that includes 32 ORFs and one *hr*, which range from Lyxy110 (Ld145) to Lyxy141 (Ld116) and are inverted compared to those of LdMNPV (Fig. 2a; Fig. 5). The LyxyMNPV genome is 4702 bp smaller than the LdMNPV genome and contains six fewer ORFs. LyxyMNPV contains six ORFs that are absent in LdMNPV (Table 1), whereas LdMNPV contains 22 ORFs that are absent in LyxyMNPV, namely *rr1, rr2a*, and *ctl-1*, as mentioned previously (see Additional file 3). LyxyMNPV and LdMNPV share 14 ORFs (Lyxy7, Lyxy8, Lyxy9, Lyxy22, Lyxy25, Lyxy30, Lyxy45, Lyxy46, Lyxy65, Lyxy73, Lyxy124, Lyxy136, Lyxy144 and Lyxy157) with unknown functions, and the homologues of these genes are not present in the other baculoviruses. These shared genes range from 50 to 370 aa in LyxyMNPV, and 10 of them contain recognisable promoters. It is possible that these genes are host range genes that are involved specifically in LyxyMNPV or other NPV infection in LD cells or other *Lymantria *species. There are 151 ORFs in common between LyxyMNPV and LdMNPV, and their order is mostly identical. Several of these ORFs are of different lengths, as shown in Additional file 4. These genes include *p74*, *dbp1*, *bro-c *to *-f*, *bro-m*, *vp91/p95*, *vp80*, *egt*, *pif-2*, *arif-1 *and *odv-e27 *as well as other genes of unassigned functions. The *hrs *of LyxyMNPV are not all in the same position and contain different numbers and orientations of repeat units compared to LdMNPV. The differences in gene content, ORF length and *hr *are possible candidates for regulators of the different degree of virulence exhibited by the two similar species [68], and it might be the same case between LyxyMNPV and LdMNPV, and even LdMNPV-like virus.

There are three Lymantriidae-derived NPV species, including LyxyMNPV, LdMNPV and OpMNPV, which have a common in vitro host cell, the LD cell line [16,17,19,23]. Comparing further the LyxyMNPV gene content to those of LdMNPV and OpMNPV, we found that these three NPVs shared 99 ORFs (Additional file 2); three of these genes were absent in the AcMNPV genome, namely Lyxy116/Op35/Ld139 (iap-3), Lyxy137/Op34/Ld120 (rr2b), and Lyxy141/Op31/Ld116 (dutpase). Of these three genes, iap-3 is one of the baculovirus genes that affect the viral host range and prevent apoptosis in baculovirus-infected cells [54]. In OpMNPV, op-iap3 could rescue AcMNPV mutants lacking p35 from induced LD cell apoptosis [56]. Silencing of op-iap3 during the OpMNPV infection of LD cells induces apoptosis [69]. These data support a possibly important role for iap-3 during the early stage of baculovirus infection in LD cells.

However, LdMNPV and OpMNPV have 22 and 55 genes, respectively, with no homologues in the LyxyMNPV genome. Among them, an important gene, hrf-1 (Ld67 and Op143), that is involved in baculoviral host range and infectivity was not found in the LyxyMNPV genome (Table 1). Studies on hrf-1 revealed that it could promote NPV infectivity (including SeMNPV, HycuNPV, BmNPV, and AcMNPV) of LD cells [69], and recombinant AcMNPV bearing *hrf-1 *also exhibited increased infectivity towards Helicoverpa zea and L. dispar larvae [70,71]. A new NPV species, MaviMNPV, which also lacks the hrf-1 gene, could infect LD cells with a low infection rate (<1%) by its egfp-recombinant virus, but it could not infect LY cells [24].

Baculovirus host range likely involves a complicated array of viral and cellular factors. However, through data from genomic analyses, we speculate that hrf-1 maybe more important for group-I NPVs during infection in LD cells than group-II NPVs. In addition, iap-3 from LyxyMNPV, LdMNPV and OpMNPV may be an important factor during NPV infection in LD cells.

## Conclusion

In conclusion, LyxyMNPV showed a high degree of colinearity and sequence identity with LdMNPV. However, these two viruses came from different geographical locations. The results of our previous studies and previous *in vitro *infection assay revealed that LyxyMNPV could be propagated in both LY and LD cell lines. Furthermore, the genome sequence analysis revealed that LyxyMNPV lacks *hrf-1*. Thus, the genes that are involved in the host range expansion of LyxyMNPV and LdMNPV are very interesting and worth further study. LyxyMNPV was highly virulent to *L. xylina *larvae, which suggests that it could be a promising agent for inclusion in I.P.M. programs for the biological control of *L. xylina *in Taiwan.

## Methods

### Insect cell lines, virus and viral DNA

Wild-type LyxyMNPV was isolated from infected larvae in Taiwan [16,17], and a LyxyMNPV clone (Ly-5) was isolated using the *L. xylina *cell line [NTU-LY-1 cells (LY) [18]] and used in this study. Viral occlusion bodies (OBs) and viral DNA were determined and prepared following the protocol described by Summers & Smith [72]. The quantity and quality of extracted DNA were determined spectrophotometrically and by electrophoresis in 1% agarose. LY cell line was cultured in TNM-FH medium [73] at 28°C. The medium contained 8% foetal bovine serum (FBS) supplemented with 50 IU/ml penicillin, 50 μg/ml streptomycin, and 1.25 μg/ml fungizone.

### Nucleotide sequence determination

The LyxyMNPV genome was sequenced to six-fold coverage by a shotgun approach. The viral DNA was sheared by hydrodynamic shearing forces into fragments with an average size of 2000 bp (HybroShear; GeneMachines). DNA fragments were size fractionated by gel electrophoresis and cloned into the EcoRV site of pBluescript II SK (-) (Stratagene). The cloned plasmids were transformed into *Escherichia coli *BH10B (Invitrogen) and the recombinant bacterial colonies were grown on LB agar containing ampicillin, X-gal and IPTG. The DNA templates were prepared using the 96-well plasmid preparation method and the sequencing was performed by using KS/SK primer set (Stratagene) and ABI 3730 DNA analyser (Applied Biosystems), and the data was compiled into contigs using the PHRED/PHRAP software package [74,75]. The assembled sequences were then edited and completed using the Sun workstation interface [76].

### DNA sequence analysis

Open reading frames (ORFs) were identified using GeneWorks software (IntelliGenetics, Inc.) and ORF Finder http://www.ncbi.nlm.nih.gov/gorf/gorf.html[77]. The criterion for defining an ORF was a size of at least 150 nt (50 aa) with minimal overlap. Promoter motifs present upstream of the putative ORFs were screened. To screen the early promoter motifs, the conserved pattern is a TATA-box motif with a cap-site CAKT of 20-30 bp located downstream within 180 bp of the initiation codon [28,78] and DTAAG within 120 bp of the initiation codon is a conserved motif of the late promoter motif [28,29]. In addition, the genome was checked in detail for the presence of any ORFs identified for AcMNPV [22], MaviMNPV [24], LdMNPV [14], SeMNPV [30] and CpGV [31] in GenBank. Homology searches were done through the National Centre for Biotechnology Information (NCBI) website using BLAST [79]. Multiple alignments and percentage identities of all LyxyMNPV ORFs with their homologues in selected genomes were generated using CLUSTAL_X [80]. The Tandem Repeats Finder http://tandem.bu.edu/trf/trf.html[81] was used to locate and analyse the homologous regions (*hrs*). GeneParityPlot analysis was performed on the LyxyMNPV genome versus the genomes of AcMNPV [22], MaviMNPV [24], LdMNPV [14], SeMNPV [30] and CpGV [31] as described previously [82] and AcMNPV were renumbered manually, starting with the *polh *gene as number one.

### Phylogenetic analysis

The phylogenetic tree was inferred from a data set of combined amino acid sequences of the 29 baculovirus core genes [22,6] of the 46 baculoviruses that were completely sequenced at the time of analysis (Additional file 1). NJ and MP analyses were performed using *MEGA*, version 4.0 [83]. *Culex nigripalpus *NPV (CuniNPV) was selected as the out-group. Bootstrap analyses were performed to evaluate the robustness of the phylogenies using 1000 replicates for both NJ and MP analyses.

## Authors' contributions

YSN carried out the molecular cloning, analysed these sequences and drafted the manuscript. MFT, TCW and YRC purified the virus genomic DNA and analysed the sequences, CYW, WHL, CFL and CHW carried out the design and draft of the manuscript. All authors read and approved the final manuscript.

## Supplementary Material

Additional file 1 **Characteristics of baculovirus genomes**. This file lists the characteristics of baculovirus genomes, including virus name, genomic size, G+C content, coding regsion, number of ORFs, *hrs *and *bro *and GeneBank assession number. Click here for file

Additional file 2 **ORFs predicted in the genome of LyxyMNPV**. This file lists the ORFs predicted in the genome of LyxyMNPV. Click here for file

Additional file 3 **LdMNPV and OpMNPV ORFs with no homologue in LyxyMNPV genome**. This file lists the LdMNPV and OpMNPV ORFs with no homologue in LyxyMNPV genome. Click here for file

Additional file 4 **The difference in the number of amino acids in the LyxyMNPV genome compared with the LdMNPV genome. Bars below zero show smaller ORFs in LyxyMNPV compared to LdMNPV**. This file shows the difference in the number of amino acids in the LyxyMNPV genome compared with the LdMNPV genome. Click here for file
