# Supplementary material for: Genomic sequencing and analyses of Lymantria xylina multiple nucleopolyhedrovirus
Source: BMC Genomics. 2010 Feb 18;11:116. doi: 10.1186/1471-2164-11-116 (PMC2830988; doi:10.1186/1471-2164-11-116)
Supplement: Additional file 1 — Characteristics of baculovirus genomes. This file lists the characteristics of baculovirus genomes, including virus name, genomic size, G+C content, coding regsion, number of ORFs, hrs and bro and GeneBank assession number. [file 1471-2164-11-116-S1.DOC]

**Additional file 1.** Characteristics of baculovirus genomes

| **Group** | **Virus name*§** | **Size (bp)** | **G+C content (%)** | **% Coding** | **No. ORFs** | **No. *hrs*** | **No. bro** | **GeneBank assession no.** |
| --- | --- | --- | --- | --- | --- | --- | --- | --- |
| **Unclassified NPV** | AgipMNPV | 155122 | 48 | 90 | 163 | 7 | 5 | NC_011345 |
|  | HearNPV | 154169 | 40 | 90 | 162 | 4 | 6 | NC_011615 |
| **Lepidopteran NPV-I** | **AcMNPV** | 133894 | 41 | 91 | 154 | 9 | 1 | NC_001623 |
|  | AgMNPV | 132239 | 44.5 | 91 | 152 | 9 | 8 | NC_008520 |
|  | AnpeNPV | 126629 | 53 | 90 | 147 | 2 | 2 | NC_008035 |
|  | AnpeNPV (L2) | 126246 | 53.5 | 97 | 145 | 6 | 2 | EF207986 |
|  | BmNPV | 128413 | 40.4 | 90 | 136 | 7 | 5 | NC_001962 |
|  | CfMNPV | 129593 | 50.1 | 90 | 146 | 5 | 1 | NC_004778 |
|  | CfDEFNPV | 131160 | 45.8 | 90 | 149 | 13 | 4 | NC_005137 |
|  | EppoNPV | 118584 | 40.7 | 92 | 136 | 5 | 1 | NC_003083 |
|  | HycuNPV | 132959 | 45 | 88 | 148 | 6 | 5 | NC_007767 |
|  | **MaviMNPV** | 111953 | 38.6 | 92 | 126 | 5 | - | NC_008725 |
|  | **OpMNPV** | 131990 | 55 | 89 | 152 | 7 | 2 | NC_001875 |
|  | PlxyNPV | 134417 | 40 | 91 | 152 | 9 | 1 | NC_008349 |
|  | RoMNPV | 131526 | 39.1 | 93 | 146 | 9 | - | NC_004323 |
|  | SfNPV | 131330 | 40 | 91 | 142 | 8 | 1 | NC_009011 |
| **Lepidopteran NPV-II** | AdhoNPV | 113220 | 35.6 | 92 | 125 | 4 | 4 | NC_004690 |
|  | AdorMNPV | 111742 | 35 | 92 | 121 | 4 | 3 | NC_011423 |
|  | AgseNPV | 157544 | 45.7 | 89 | 153 | 5 | 4 | NC_007921 |
|  | ChchNPV | 149622 | 39.1 | 89 | 151 | - | 4 | NC_007151 |
|  | ClbiNPV | 135454 | 37 | 84 | 129 | - | 3 | NC_008293 |
|  | EcobNPV | 131204 | 37 | 89 | 126 | 3 | 2 | NC_008586 |
|  | EupsNPV | 141291 | 40 | 87 | 139 | 4 | 2 | NC_012639 |
|  | Hear NPV (G4) | 131403 | 39 | 86 | 135 | 5 | 3 | NC_002654 |
|  | HearNPV (C1) | 130759 | 38.9 | 87 | 134 | 5 | 3 | NC_003094 |
|  | HearSNPV | 132425 | 39 | 87 | 143 | 5 | 4 | NC_011354 |
|  | HzSNPV | 130869 | 39.1 | 87 | 139 | 5 | 3 | NC_003349 |
|  | **LdMNPV** | 161046 | 57.5 | 86 | 163 | 13 | 16 | NC_001973 |
|  | **LyxyMNPV** | **156344** | **53.5** | **-** | **157** | **13** | **14** | **GQ202541** |
|  | LsNPV | 168041 | 48 | 83 | 169 | 8 | 10 | NC_008348 |
|  | MacoNPV (A) | 155060 | 41.7 | 90 | 169 | 4 | 8 | NC_003529 |
|  | MacoNPV (B) | 158482 | 40.0 | 89 | 168 | 4 | 7 | NC_004117 |
|  | OrleNPV | 156179 | 39 | 79 | 135 | 6 | 5 | NC_010276 |
|  | **SeMNPV** | 135611 | 44 | 88 | 139 | 6 | - | NC_002169 |
|  | SpltNPV | 139342 | 42.7 | 88 | 141 | 17 | 2 | NC_003102 |
|  | SpltNPV (II) | 148634 | 44 | 83 | 147 | 7 | 2 | NC_011616 |
|  | TnSNPV | 134394 | 39.0 | 90 | 144 | - | 2 | NC_007383 |
| **Lepidopteran GV** | AdorGV | 99657 | 34.5 | 92 | 119 | - | - | NC_005038 |
|  | AgseGV | 131680 | 37.2 | 88 | 132 | - | - | NC_005839 |
|  | ChocGV | 104710 | 32.7 | 87 | 116 | 5 | - | NC_008168 |
|  | **CpGV** | 123500 | 43.2 | 88 | 143 | - | 1 | NC_002816 |
|  | CrleGV | 110907 | 32.4 | 89 | 129 | 3 | - | NC_005068 |
|  | HearGV | 169794 | 40 | 88 | 179 | 9 | 10 | NC_010240 |
|  | PhopGV | 119217 | 35.7 | 85 | 130 | 12 | 1 | NC_004062 |
|  | PlxyGV | 100999 | 40.7 | 88 | 120 | 4 | - | NC_002593 |
|  | SpltGV | 124121 | 38 | 87 | 136 | - | 6 | NC_009503 |
|  | XecnGV | 178733 | 40.7 | 87 | 181 | 9 | 7 | NC_002331 |
| **Hymenopteran NPV** | NeabNPV | 84264 | 33.5 | 88 | 93 | - | - | NC_008252 |
|  | NeleNPV | 81755 | 33.3 | 88 | 89 | - | - | NC_005906 |
|  | NeseNPV | 86462 | 33.8 | 84 | 90 | 6 | - | NC_005905 |
| **Dipteran NPV** | CuniNPV | 108252 | 50.9 | 88 | 109 | 4 | 6 | NC_003084 |

*****Virus name with underline were not used for phylogenetic tree construction.

**§**Virus name printed in bold were used for further comparison.
