# Supplementary material for: Genomic sequencing and analyses of Lymantria xylina multiple nucleopolyhedrovirus
Source: BMC Genomics. 2010 Feb 18;11:116. doi: 10.1186/1471-2164-11-116 (PMC2830988; doi:10.1186/1471-2164-11-116)
Supplement: Additional file 2 — ORFs predicted in the genome of LyxyMNPV. This file lists the ORFs predicted in the genome of LyxyMNPV. [file 1471-2164-11-116-S2.DOC]

**Additional file 2.** ORFs predicted in the genome of LyxyMNPV.

| **ORF** | **Name*** | **Position†** | **Length**  **(aa)** | **Promoter**  **motifs‡** | **Homologoues (% identity) §** | | | | | | |
| --- | --- | --- | --- | --- | --- | --- | --- | --- | --- | --- | --- |
|  |  |  |  |  | **AcMNPV** | **OpMNPV** | **MaviMNPV** | **LdMNPV** | **SeMNPV** | **CpGV** | **Other** |
| 1 | *polh* | 1→738 | 245 | L | Ac8 (80) 245 | Op3 (79) 244 | Mv1 (78) 245 | Ld1 (100) 245 | Se1 (81) 246 | Cp1 (54) 248 |  |
| 2 | *orf1629* | 746←2266 | 506 | - | Ac9 (16) 543 | Op2 (11) 474 | Mv2 (17) 539 | Ld2 (71) 555 | Se2 (10) 462 |  |  |
| 3 | *pk-1* | 2268→3092 | 274 | L | Ac10 (37) 272 | Op1 (39) 274 | Mv3 (38) 270 | Ld3 (88) 274 | Se3 (40) 295 | Cp3 (28) 279 |  |
| 4 | *mucin-like* | 3613←6777 | 1054 | L |  |  |  | Ld4 (22) 1029 |  |  |  |
| 5 |  | 6511→7023 | 170 | e |  |  |  | Ld5 (15) 189 |  |  |  |
| 6 | *g22* | 7061→7603 | 180 | e |  |  |  | Ld7 (81) 191 |  |  |  |
|  | *hr2* (4 repeats) | 7850 ~ 8774 |  |  |  |  |  | *hr2* (4 repeats) |  |  |  |
| 7 |  | 8809→9492 | 227 | E, L |  |  |  | Ld9 (26) 172 |  |  |  |
| 8 |  | 9792→10616 | 274 | E |  |  |  | Ld11 (18) 244 |  |  |  |
| 9 |  | 11093→11950 | 285 | E |  |  |  | Ld12 (22) 172 |  |  |  |
| 10 | ***odv-e56*** | 12265→13335 | 356 | E, L | Ac148 (51) 376 | Op146 (51) 374 | Mv116 (50) 378 | Ld14 (87) 356 | Se6 (47) 371 | Cp18 (43) 355 |  |
| 11 |  | 13618→13794 | 58 | - |  |  |  |  |  |  |  |
| 12 | *ie-1* | 13821←15530 | 569 | E | Ac147 (21) 582 | Op145 (23) 560 | Mv115 (22) 575 | Ld15 (81) 566 | Se132 (20) 714 | Cp7 (8) 488 |  |
| 13 |  | 15590→16210 | 206 | L | Ac146 (31) 201 | Op144 (28) 197 | Mv114 (26) 225 | Ld16 (80) 208 | Se133 (34) 200 | Cp8 (19) 192 |  |
| 14 |  | 16263←16541 | 92 | L | Ac145 (35) 77 | Op142 (42) 95 | Mv113 (44) 95 | Ld17 (85) 92 | Se134 (54) 92 | Cp9 (31) 101 |  |
| 15 | ***odv-e27*** | 16552←17412 | 286 | L | Ac144 (46) 290 | Op141 (46) 297 | Mv112 (46) 288 | Ld18 (84) 283 | Se135 (58) 281 | Cp97 (22) 288 |  |
| 16 | *odv-e18* | 17429←17695 | 88 | - | Ac143 (32) 62 | Op140 (44) 85 | Mv111 (44) 89 | Ld19 (98) 88 | Se136 (45) 80 | Cp14 (32) 84 |  |
| 17 | ***p49*** | 17667←19118 | 483 | e, L | Ac142 (46) 94 | Op139 (45) 484 | Mv110 (47) 477 | Ld20 (93) 483 | Se137 (49) 460 | Cp15 (27) 457 |  |
| 18 | *ie-0* | 19115←19891 | 258 | E | Ac141 (25) 261 | Op138 (24) 245 | Mv109 (25) 255 | Ld21 (89) 258 | Se138 (31) 244 |  |  |
| 19 |  | 20088→20255 | 55 | - |  |  |  |  |  |  |  |
| 20 | *dna ligase* | 20257→22008 | 583 | - |  |  |  | Ld22 (72) 548 |  | Cp120 (18) 570 |  |
| 21 | *me53* | 22189→23298 | 369 | - | Ac139 (17) 449 | Op137 (10) 455 | Mv107 (16) 465 | Ld23 (81) 342 | Se7 (27) 390 | Cp143 (14) 303 |  |
| 22 |  | 23551→24018 | 155 | E |  |  |  | Ld25 (74) 154 |  |  |  |
| 23 |  | 24110←24328 | 72 | E |  |  |  | Ld26 (80) 72 |  |  |  |
| 24 | ***p74*** | 24340→26349 | 596 | - | Ac138 (47) 645 | Op134 (56) 644 | Mv106 (45) 645 | Ld27 (84) 672 | Se131 (49) 653 | Cp60 (31) 688 |  |
| 25 |  | 26397→27509 | 370 | E |  |  |  | Ld28 (89) 379 |  |  |  |
|  | *hr3a* (7 repeats) | 27643 ~ 28204 |  |  |  |  |  | *hr3a* (7 repeats) |  |  |  |
| 26 |  | 28260→28691 | 143 | - | Ac4 (13) 151 | Op8 (13) 146 |  | Ld29 (83) 146 |  |  |  |
| 27 |  | 28786→29070 | 94 | L | Ac150 (25) 99 |  | Mv118 (16) 113 | Ld30 (90) 94 | Se96 (11) 113 | Cp79 (8) 156 |  |
|  | *hr3b* (4 repeats) | 29248 ~ 29837 |  |  |  |  |  | *hr3b* (4 repeats) |  |  |  |
| 28 | *bro-a* | 29843→30895 | 350 | - |  |  |  | Ld32 (87) 350 |  |  |  |
|  | *hr3c* (3 repeats) | 30880 ~ 31519 |  |  |  |  |  | *hr3c* (3 repeats) |  |  |  |
| 29 | *bro-b* | 31591→32490 | 299 | - | Ac2 (39) 328 |  |  | Ld33 (81) 323 |  |  |  |
| 30 |  | 32622←33383 | 253 | - |  |  |  | Ld34 (90) 253 |  |  |  |
| 31 |  | 33501←34487 | 328 | e, L | Ac11 (28) 340 | Op11 (26) 331 | Mv4 (28) 332 | Ld35 (81) 359 |  |  |  |
| 32 |  | 34517←34888 | 123 | e, L | Ac26 (27) 129 | Op42 (27) 127 | Mv17 (29) 129 | Ld36 (87) 123 | Se125 (23) 135 |  |  |
| 33 | *dbp* | 34851→35648 | 265 | e | Ac25 (13) 316 | Op43 (16) 300 | Mv16 (13) 312 | Ld37 (75) 239 | Se126 (14) 328 | Cp81 (8) 290 |  |
| 34 | *lef-6* | 35650→36144 | 164 | e, L | Ac28 (20) 173 | Op40 (22) 138 | Mv19 (19) 172 | Ld38 (64) 159 | Se127 (26) 163 | Cp80 (13) 101 |  |
| 35 | *ac29* | 36283←36489 | 69 | - | Ac29 (29) 71 | Op39 (30) 75 | Mv20 (39) 67 | Ld39 (94) 68 | Se128 (25) 136 | Cp19 (17) 75 |  |
| 36 | *p26* | 36574→37326 | 250 | L | Ac136 (17) 240 | Op132 (18) 230 | Mv104 (17) 240 | Ld40 (76) 253 | Se129 (27) 278 |  |  |
| 37 |  | 36750→37595 | 281 | e, L | Ac137 (5) 94 | Op133 (5) 92 | Mv105 (6) 73 | Ld41 (23) 77 | Se130 (13) 88 |  |  |
| 37’ | *p10 in orf37* | 37362→37595 | 77 | L | Ac137 (19) 94 | Op133 (26) 92 | Mv105 (25) 73 | Ld41 (87) 77 | Se130 (45) 88 |  |  |
| 38 |  | 37596←38165 | 189 | L | Ac34 (25) 215 | Op26 (29) 209 | Mv23 (25) 212 | Ld42 (86) 188 | Se124 (39) 187 |  |  |
| 39 | *ubiquitin* | 38244→38699 | 151 | L | Ac35 (35) 77 | Op25 (43) 93 | Mv24 (36) 77 | Ld43 (81) 150 | Se123 (40) 80 | Cp54 (41) 94 |  |
| 40 | *39K* | 38893←39696 | 267 | - | Ac36 (26) 275 | Op24 (29) 261 | Mv25 (26) 266 | Ld44 (84) 264 | Se120 (29) 317 | Cp57 (7) 241 |  |
| 41 | *lef-11* | 39482←40057 | 191 | e | Ac37 (19) 112 | Op23 (20) 125 | Mv26 (21) 111 | Ld45 (80) 187 | Se119 (23) 103 | Cp58 (15) 134 |  |
| 42 | *ac38* | 39967←40716 | 249 | L | Ac38 (45) 216 | Op22 (50) 209 | Mv27 (44) 216 | Ld46 (90) 247 | Se118 (52) 261 | Cp69 (36) 220 |  |
| 43 | *dbp1* | 40931→41851 | 307 | - | Ac25 (21) 316 | Op43 (22) 300 | Mv16 (20) 312 | Ld47 (80) 257 | Se126 (35) 328 | Cp81 (11) 290 |  |
| 44 | ***p47*** | 42143←43315 | 390 | L | Ac40 (52) 401 | Op45 (52) 399 | Mv29 (51) 399 | Ld48 (96) 390 | Se115 (65) 400 | Cp68 (37) 460 |  |
|  | *hr4* (3 repeats) | 43380 ~ 43635 |  |  |  |  |  | *hr4* (5repeats) |  |  |  |
| 45 |  | 43746→44105 | 119 | e |  |  |  | Ld49 (17) 106 |  |  |  |
| 46 |  | 44298→44450 | 50 | e |  |  |  | Ld49 (13) 106 |  |  |  |
| 47 | *helicase-2* | 44527→45906 | 459 | e, L |  |  |  | Ld50 (98) 460 |  | Cp126 (50) 457 |  |
| 48 | ***lef-8*** | 46066←48675 | 869 | - | Ac50 (59) 876 | Op54 (57) 884 | Mv35 (59) 878 | Ld51 (93) 874 | Se112 (62) 906 | Cp131 (47) 873 |  |
| 49 |  | 48674→49591 | 305 | e | Ac51 (12) 318 | Op55 (11) 318 | Mv36 (11) 318 |  | Se111 (13) 415 |  | Clbi31 (15) 340 |
| 50 |  | 49626←50171 | 181 | - | Ac52 (13) 123 |  | Mv37 (25) 194 | Ld53 (45) 300 | Se109 (23) 162 |  |  |
| 51 |  | 50174←50326 | 50 | e | Ac52 (5) 123 |  | Mv37 (3) 194 | Ld53 (13) 300 | Se109 (2) 162 |  |  |
| 52 |  | 50217→50645 | 142 | e, L | Ac53 (40) 139 | Op56 (38) 146 | Mv38 (40) 139 | Ld54 (91) 142 | Se108 (55) 137 | Cp134 (15) 133 |  |
| 53 |  | 50695←51744 | 349 | e, L |  |  |  | Ld55 (60) 361 | Se107 (20) 344 |  |  |
| 54 | *lef-10* | 51922→52176 | 84 | L | Ac53a (36) 78 | Op57 (21) 80 | Mv39 (38) 78 | Ld56 (84) 76 | Se106 (45) 77 | Cp137 (23) 89 |  |
| 55 | ***vp1054*** | 52037→53035 | 332 | L | Ac54 (36) 365 | Op58 (31) 378 | Mv40 (38) 362 | Ld57 (91) 332 | Se105 (52) 346 | Cp138 (26) 332 |  |
| 56 |  | 53032→53346 | 104 | - | Ac55 (15) 73 | Op59 (16) 68 | Mv41 (17) 69 | Ld58 (55) 64 | Se104 (25) 67 |  |  |
| 57 |  | 53468→54070 | 200 | e, E | Ac57 (33) 161 | Op61 (32) 163 |  | Ld60 (70) 164 | Se102 (38) 178 |  |  |
| 58 | *ac58-59* | 54053←54553 | 166 | L | Ac59 (27) 69 | Op62 (27) 176 | Mv43 (25) 161 | Ld61 (56) 189 | Se101 (35) 195 |  |  |
| 59 |  | 54643←54930 | 95 | L | Ac60 (38) 87 | Op63 (36) 90 | Mv44 (38) 82 | Ld62 (83) 95 | Se100 (44) 89 |  |  |
| 60 | *fp25k* | 55029←55682 | 217 | L | Ac61 (52) 214 | Op64 (47) 208 | Mv45 (49) 209 | Ld63 (81) 217 | Se98 (52) 195 | Cp118 (22) 161 |  |
| 61 | ***lef-9*** | 55787→57277 | 496 | - | Ac62 (63) 516 | Op65 (64) 489 | Mv46 (66) 490 | Ld64 (95) 496 | Se97 (67) 495 | Cp117 (53) 499 |  |
| 62 | *vef-1* | 57327→59675 | 782 | e, L |  |  |  | Ld65 (89) 783 |  |  |  |
| 63 | *ctl-2* | 59618←59779 | 53 | L | Ac3 (46) 53 | Op30 (66) 52 |  | Ld66 (40) 92 |  |  |  |
| 64 | *gp37* | 60227←61039 | 270 | e, L | Ac64 (41) 155 | Op69 (41) 321 | Mv48 (41) 287 | Ld68 (92) 269 | Se25 (56) 267 | Cp13 (37) 251 |  |
| 65 |  | 61080→61442 | 120 | e |  |  |  | Ld69 (7) 50 |  |  |  |
| 66 | *chitinase* | 61482←63200 | 572 | - | Ac126 (63) 551 | Op124 (65) 550 | Mv95 (62) 551 | Ld70 (95) 528 | Se19 (59) 572 | Cp10 (52) 594 |  |
| 67 | *bro-c* | 63413←63877 | 154 | - |  |  |  | Ld71 (27) 528 |  |  |  |
| 68 | *bro-d* | 63886←64878 | 330 | L | Ac2 (19) 328 |  |  | Ld71 (39) 528 |  |  | Ls52 (44) 485 |
| 69 | *bro-e* | 65022→65612 | 196 | - |  |  |  | Ld71 (21) 528 |  |  |  |
| 70 | *bro-f* | 65593→66342 | 249 | L |  |  |  | Ld72 (17) 510 |  |  | TnS108 (24) 490 |
| 71 | *bro-g* | 66445→67176 | 243 | e |  |  |  | Ld75 (75) 222 |  |  |  |
| 72 |  | 66630→67772 | 380 | e, L | Ac111 (12) 67 | Op112 (6) 72 |  | Ld76 (18) 87 |  |  |  |
| 73 |  | 67414→68040 | 208 | - |  |  |  | Ld77 (87) 214 |  |  |  |
| 74 | *v-cath* | 68233→69240 | 335 | e, L | Ac127 (64) 323 | Op125 (60) 324 | Mv96 (63) 324 | Ld78 (87) 356 | Se16 (52) 337 | Cp11 (40) 333 |  |
| 75 | *iap-2* | 69237←69923 | 228 | L | Ac71 (18) 249 | Op74 (28) 236 | Mv54 (28) 246 | Ld79 (76) 234 | Se88 (7) 317 | Cp94 (18) 243 |  |
| 76 | ***ac68*** | 69920←70312 | 130 | - | Ac68 (24) 192 | Op73 (33) 131 | Mv52 (23) 184 | Ld80 (84) 128 | Se90 (37) 133 | Cp114 (22) 126 |  |
| 77 | *lef-3* | 70311→71444 | 377 | e | Ac67 (12) 385 | Op72 (20) 373 | Mv51 (13) 372 | Ld81 (40) 374 | Se91 (15) 422 | Cp113 (4) 353 |  |
| 78 | *desmop* | 71540←73933 | 797 | e | Ac66 (16) 808 | Op71 (16) 875 | Mv50 (15) 766 | Ld82 (71) 778 | Se92 (19) 704 | Cp112 (10) 718 |  |
| 79 | ***dna-pol*** | 73815→76886 | 1023 | e | Ac65 (41) 984 | Op70 (40) 985 | Mv49 (40) 980 | Ld83 (88) 1014 | Se93 (51) 1063 | Cp111 (29) 1051 |  |
| 80 | *ac75* | 77020←77406 | 128 | L | Ac75 (23) 133 | Op78 (23) 130 | Mv58 (22) 133 | Ld84 (88) 128 | Se94 (51) 129 | Cp108 (16) 148 |  |
| 81 | *ac76* | 77408←77668 | 86 | L | Ac76 (39) 84 | Op79 (39) 84 | Mv59 (38) 84 | Ld85 (97) 86 | Se95 (72) 85 | Cp107 (30) 84 |  |
| 82 | ***vlf-1*** | 77718←78848 | 376 | L | Ac77 (63) 379 | Op80 (64) 374 | Mv60 (62) 383 | Ld86 (90) 378 | Se82 (61) 372 | Cp106 (29) 378 |  |
| 83 | *ac78* | 78862←79194 | 110 | e, L | Ac78 (23) 109 | Op81 (30) 105 | Mv61 (25) 106 | Ld87 (68) 113 | Se81 (31) 127 | Cp105 (9) 111 |  |
| 84 | ***gp41*** | 79191←80144 | 317 | L | Ac80 (46) 409 | Op83 (44) 367 | Mv63 (45) 406 | Ld88 (95) 323 | Se80 (51) 331 | Cp104 (29) 289 |  |
| 85 | ***ac81*** | 80125←80784 | 219 | - | Ac81 (47) 233 | Op84 (47) 218 | Mv64 (45) 227 | Ld89 (86) 219 | Se79 (43) 240 | Cp103 (36) 191 |  |
| 86 | *tlp20* | 80681←81247 | 188 | L | Ac82 (27) 180 | Op85 (24) 155 | Mv65 (24) 176 | Ld90 (70) 223 | Se78 (41) 196 | Cp102 (10) 216 |  |
| 87 | ***vp91/p95*** | 81216→83654 | 812 | L | Ac83 (38) 847 | Op86 (38) 819 | Mv66 (36) 843 | Ld91 (78) 864 | Se77 (42) 813 | Cp101 (14) 665 |  |
|  | *hr5* (5 repeats) | 83697 ~ 84470 |  |  |  |  |  | *hr5* (7 repeats) |  |  |  |
| 88 | ***vp39*** | 84595←85632 | 345 | L | Ac89 (37) 347 | Op90 (46) 351 | Mv67 (37) 345 | Ld92 (88) 352 | Se75 (50) 326 | Cp96 (26) 285 |  |
| 89 | ***lef-4*** | 85631→87088 | 485 | - | Ac90 (40) 464 | Op91 (40) 457 | Mv68 (39) 464 | Ld93 (86) 485 | Se74 (43) 466 | Cp95 (27) 480 |  |
| 90 | ***p33*** | 87120←87875 | 251 | e, L | Ac92 (49) 259 | Op93 (46) 282 | Mv69 (49) 256 | Ld94 (88) 251 | Se73 (55) 252 | Cp93 (32) 251 |  |
| 91 | *ac93* | 87874→88356 | 160 | - | Ac93 (43) 161 | Op94 (40) 159 | Mv70 (42) 162 | Ld95 (95) 159 | Se72 (62) 157 | Cp92 (30) 161 |  |
| 92 | *odv-e25* | 88358→89011 | 217 | e | Ac94 (42) 228 | Op95 (42) 229 | Mv71 (42) 229 | Ld96 (94) 217 | Se71 (65) 216 | Cp91 (55) 213 |  |
| 93 | ***helicase*** | 89173←92814 | 1213 | e, L | Ac95 (39) 1221 | Op96 (35) 1223 | Mv72 (38) 1224 | Ld97 (91) 1218 | Se70 (46) 1222 | Cp90 (20) 1131 |  |
| 94 | ***19kda*** | 92771→93292 | 173 | e, L | Ac96 (44) 175 | Op97 (43) 172 | Mv73 (43) 173 | Ld98 (91) 173 | Se69 (61) 170 | Cp89 (32) 161 |  |
| 95 | ***38K*** | 93286←94233 | 315 | L | Ac98 (40) 320 | Op99 (41) 313 | Mv75 (38) 321 | Ld99 (90) 322 | Se67 (48) 300 | Cp88 (31) 343 |  |
| 96 | ***lef-5*** | 94126→94962 | 278 | L | Ac99 (45) 265 | Op100 (43) 263 | Mv76 (46) 265 | Ld100 (90) 278 | Se66 (50) 279 | Cp87 (37) 242 |  |
| 97 | *p6.9* | 94956←95258 | 100 | L | Ac100 (35) 55 | Op101 (33) 51 | Mv77 (35) 55 | Ld101 (85) 99 | Se65 (34) 75 | Cp86 (23) 49 |  |
| 98 | *p40* | 95375←96520 | 381 | L | Ac101 (36) 361 | Op102 (38) 354 | Mv78 (37) 360 | Ld102 (91) 381 | Se64 (43) 388 | Cp85 (19) 380 |  |
| 99 | *p12* | 96537←96902 | 121 | e, L | Ac102 (28) 122 | Op103 (24) 112 | Mv79 (28) 114 | Ld103 (95) 121 | Se63 (27) 106 | Cp84 (13) 109 |  |
| 100 | *p45* | 96895←98064 | 389 | L | Ac103 (40) 387 | Op104 (35) 411 | Mv80 (40) 387 | Ld104 (91) 389 | Se62 (51) 375 | Cp83 (27) 439 |  |
| 101 | *vp80* | 98086→100785 | 899 | e | Ac104 (12) 691 | Op105 (12) 624 | Mv81 (12) 686 | Ld105 (69) 964 | Se61 (10) 556 |  |  |
| 102 |  | 100787→100957 | 56 | - | Ac110 (25) 56 | Op111 (34) 56 | Mv86 (24) 56 | Ld106 (89) 56 | Se60 (42) 59 |  |  |
| 103 | ***ac109*** | 100963→102066 | 367 | L | Ac109 (46) 390 | Op109 (44) 390 | Mv85 (47) 387 | Ld107 (91) 366 | Se59 (48) 356 | Cp55 (25) 326 |  |
| 104 |  | 102069→102362 | 97 | e | Ac108 (29) 105 | Op108 (23) 108 | Mv84 (27) 103 | Ld108 (77) 97 | Se58 (33) 114 |  |  |
| 105 | *ac112-113* | 102401→103402 | 333 | e | Ac112-113 (24) 258 |  |  | Ld109 (85) 336 |  |  |  |
| 106 | *pkip* | 103488→104027 | 179 | e, L | Ac24 (21) 169 | Op44 (21) 166 | Mv15 (21) 159 | Ld110 (94) 179 | Se32 (27) 164 |  |  |
| 107 |  | 104040←104333 | 97 | - |  |  |  | Ld111 (84) 100 | Se33 (17) 112 |  |  |
| 108 | *bro-h* | 104585←105832 | 415 | e | Ac2 (46) 328 | Op116 (7) 88 |  | Ld114 (90) 403 |  |  |  |
| 109 | *bro-i* | 105958←106995 | 318 |  |  |  |  | Ld32 (68) 350 |  |  | Anpe89 (78) 399 |
|  | *hr6* (4 repeats) | 106996 ~ 107442 |  |  |  |  |  |  |  |  |  |
| 110 | *sod* | 107453←107917 | 154 | - | Ac31 (74) 151 | Op29 (68) 152 | Mv22 (72) 152 | Ld145 (94) 154 | Se48 (63) 151 | Cp59 (45) 132 |  |
| 111 |  | 107995→108324 | 109 | e |  |  |  | Ld144 (78) 113 | Se49 (19) 130 |  |  |
| 112 | ***pif-3*** | 108489→109091 | 200 | L | Ac115 (41) 204 | Op115 (42) 205 | Mv89 (41) 204 | Ld143 (87) 203 | Se50 (45) 214 | Cp35 (30) 199 |  |
| 113 |  | 109100→109465 | 121 | - |  |  |  | Ld142 (70) 118 | Se51 (13) 142 |  |  |
| 114 |  | 109535→111178 | 547 | L |  |  |  | Ld141 (78) 542 | Se52 (16) 529 |  |  |
| 115 | *ac106-107* | 111204→111905 | 233 | e, L | Ac106-107 (49) 243 | Op107 (49) 256 | Mv83 (49) 238 | Ld140 (86) 246 | Se53 (49) 222 | Cp52 (16) 342 |  |
| 116 | *iap-3* | 111897←112346 | 149 | e, L |  | Op35 (22) 268 |  | Ld139 (83) 155 | Se110 (16) 313 | Cp17 (17) 275 |  |
| 117 |  | 112588←113463 | 291 | E |  |  |  | Ld138 (88) 291 | Se54 (24) 364 | Cp16 (20) 196 |  |
| 118 | ***lef-2*** | 113571←114203 | 210 | e | Ac6 (34) 210 | Op6 (38) 204 | Mv126 (34) 210 | Ld137 (81) 216 | Se12 (39) 209 | Cp41 (18) 171 |  |
| 119 | *9.7 kda* | 114169←114435 | 88 | e, L |  |  |  | Ld137a (77) 88 | Se11 (20) 105 |  |  |
| 120 | *pp34* | 114505←115437 | 310 | L | Ac131 (16) 252 | Op129 (30) 297 | Mv100 (22) 314 | Ld136 (92) 313 | Se46 (40) 335 |  |  |
| 121 |  | 115559→115894 | 111 | e, L |  |  |  | Ld135 (73) 123 |  |  |  |
| 122 | *p24* | 115854←116531 | 228 | e, L | Ac129 (30) 198 | Op127 (37) 192 | Mv98 (30) 192 | Ld133-135 (88) 223 | Se10 (45) 248 | Cp71 (19) 203 |  |
| 123 |  | 116547←116864 | 105 | E |  |  |  |  |  |  | Agse52 (35) 112 |
| 124 |  | 117076←117318 | 80 | e |  |  |  | Ld132 (62) 81 |  |  |  |
| 125 | *odv-e66* | 117386←119338 | 650 | e, L | Ac46 (35) 704 | Op50 (40) 682 |  | Ld131 (79) 665 | Se57 (39) 723 | Cp37 (37) 683 |  |
| 126 | *efp/ld130* | 119417←121441 | 674 | E, L | Ac23 (14) 690 | Op21 (15) 657 | Mv14 (15) 624 | Ld130 (96) 676 | Se8 (36) 656 | Cp31 (19) 601 |  |
| 127 |  | 121573→124200 | 875 | e |  |  |  | Ld129 (78) 884 | Se30 (25) 886 |  |  |
| 128 |  | 124423←125088 | 221 | - | Ac17 (17) 209 | Op16 (21) 207 | Mv9 (18) 215 | Ld128 (80) 226 | Se29 (24) 213 |  |  |
| 129 |  | 125096←125665 | 189 | L |  |  |  | Ld127 (79) 192 | Se28 (22) 190 |  |  |
| 130 |  | 125790←126041 | 83 | e, L |  |  |  |  |  |  |  |
| 131 | *gag-like* | 126071←128140 | 689 | - |  |  |  |  |  |  |  |
| 132 | *egt* | 128507←130027 | 506 | - | Ac15 (40) 506 | Op14 (43) 489 | Mv7 (38) 503 | Ld125 (82) 560 | Se27 (50) 523 | Cp141 (34) 484 |  |
|  | *hr7* (2 repeats) | 130131 ~ 130367 |  |  |  |  |  | *hr6* (4 repeats) |  |  |  |
| 133 |  | 130384←130785 | 133 | L |  |  |  | Ld124 (82) 133 | Se15 (30) 154 |  |  |
| 134 | ***lef-1*** | 130828→131532 | 234 | - | Ac14 (36) 266 | Op13 (38) 243 | Mv6 (34) 264 | Ld123 (84) 234 | Se14 (45) 216 | Cp74 (34) 235 |  |
| 135 | *38.7K* | 131514→132173 | 219 | - | Ac13 (15) 327 | Op12 (19) 320 | Mv5 (15) 323 | Ld122 (71) 200 | Se13 (16) 363 | Cp73 (7) 198 |  |
| 136 |  | 132160→132375 | 71 | - |  |  |  | Ld121 (18) 78 |  |  |  |
| 137 | *rr2b* | 132549←133565 | 338 | - |  | Op34 (15) 349 |  | Ld120 (89) 348 | Se45 (51) 313 |  |  |
| 138 | *pif-2* | 133595←135124 | 509 | - | Ac22 (43) 382 | Op20 (44) 382 | Mv13 (43) 382 | Ld119 (75) 407 | Se35 (48) 413 | Cp48 (36) 372 |  |
| 139 | *arif-1* | 134577→135656 | 272 | e | Ac21 (11) 319 | Op19 (12) 298 | Mv12 (10) 406 | Ld118 (64) 269 | Se34 (18) 281 |  |  |
| 140 |  | 135437→136165 | 242 | - | Ac63 (14) 155 |  | Mv47 (14) 149 | Ld117 (40) 154 |  |  |  |
| 141 | *dutpase* | 136350←136799 | 149 | - |  | Op31 (11) 317 |  | Ld116 (96) 149 | Se55 (48) 143 |  |  |
| 142 | *bro-j* | 136951→137928 | 325 | - | Ac2 (34) 328 |  |  | Ld33 (63) 323 |  |  | CfDEF59 (64) 320 |
| 143 |  | 137956←138516 | 186 | E | Ac12 (18) 217 |  |  | Ld151 (81) 168 |  |  |  |
| 144 |  | 138638→139393 | 251 | L |  |  |  | Ld152 (84) 250 |  |  |  |
| 145 | *bro-k* | 139461←140441 | 326 | - | Ac2 (36) 328 |  |  | Ld161 (74) 337 |  |  |  |
|  | *hr8a* (3 repeats) | 140560 ~ 140803 |  |  |  |  |  |  |  |  |  |
| 146 | *bro-l* | 140861←141898 | 345 | - | Ac2 (74) 328 | Op116 (11) 88 |  | Ld153 (95) 338 |  |  |  |
|  | *hr8b* (3 repeats) | 141998 ~ 142231 |  |  |  |  |  | *hr7d* (5 repeats) |  |  |  |
| 147 | *bro-m* | 142353→143777 | 474 | - |  |  |  | Ld154 (58) 336 |  |  |  |
| 148 | ***pif-1*** | 143869→145473 | 534 | L | Ac119 (45) 530 | Op119 (42) 529 | Mv91 (45) 530 | Ld155 (90) 530 | Se36 (38) 526 | Cp75 (31) 538 |  |
| 149 | *fgf* | 145808←146647 | 279 | L | Ac32 (18) 181 | Op27 (20) 205 |  | Ld156 (86) 285 | Se38 (16) 404 | Cp123 (6) 400 |  |
| 150 | ***alk-exo*** | 146680←147915 | 411 | L | Ac133 (36) 419 | Op131 (40) 424 | Mv102 (36) 418 | Ld157 (84) 420 | Se41 (41) 413 | Cp125 (31) 398 |  |
| 151 |  | 147946←149079 | 377 | - | Ac18 (14) 353 | Op17 (17) 355 | Mv10 (15) 351 | Ld158 (71) 373 | Se43 (17) 280 |  |  |
| 152 |  | 149066→149422 | 118 | e, L | Ac19 (23) 108 | Op18 (17) 104 | Mv11 (23) 109 | Ld159 (81) 118 | Se42 (22) 81 |  |  |
| 153 | *vef-2* | 149464→151830 | 788 | L |  |  |  | Ld160 (94) 788 |  |  |  |
|  | *hr8c* (3 repeats) | 151851 ~ 152261 |  |  |  |  |  | *hr8* (2 repeats) |  |  |  |
| 154 | *bro-n* | 152269→153294 | 341 | - | Ac2 (31) 328 |  |  | Ld161 (72) 337 |  |  |  |
|  | *hr9* (4 repeats) | 153376 ~ 153737 |  |  |  |  |  |  |  |  |  |
| 155 | ***odv-e27*** | 153801→154367 | 188 | - | Ac144 (18) 290 | Op141 (17) 297 | Mv112 (18) 288 | Ld18 (18) 383 | Se135 (18) 281 | Cp97 (10) 288 | Anpe135 (18) 308 |
| 156 |  | 154453←154749 | 98 | - | Ac12 (11) 217 |  |  | Ld162 (75) 91 |  |  |  |
| 157 |  | 154863→155837 | 324 | - |  |  |  | Ld163 (67) 329 |  |  |  |
|  | *hr1* (3 repeats) | 155968 ~ 156326 |  |  |  |  |  | *hr1* (6 repeats) |  |  |  |

*Baculovirus core genes are printed in bold and underlined; additional genes common to lepidopteran NPVs and GVs are underlined.

†Nucleotides in the LyxyMNPV genome were numbered sequentially, beginning with the A (position 1) of the initiation codon (ATG) of the *polh* gene, in the direction of transcription of the *polh* gene. The directions of the transcripts are indicated by arrows.

‡Promoter motifs present upstream of ORF. E, Early promoter motif [TATA box TATAWAW followed by CAKT motif 20–30 bp downstream] within 180 bp of the initiation codon; e, enhancer-like element (CGTGC) within 210 bp; L, late promoter motif DTAAG within 120 bp.

§The ORF number of a putative homologue is shown with the percent amino acid identity in parentheses, followed by the length of the homologue.
