# Supplementary material for: Genomic sequencing and analyses of Lymantria xylina multiple nucleopolyhedrovirus
Source: BMC Genomics. 2010 Feb 18;11:116. doi: 10.1186/1471-2164-11-116 (PMC2830988; doi:10.1186/1471-2164-11-116)
Supplement: Additional file 3 — LdMNPV and OpMNPV ORFs with no homologue in LyxyMNPV genome. This file lists the LdMNPV and OpMNPV ORFs with no homologue in LyxyMNPV genome. [file 1471-2164-11-116-S3.DOC]

**Additional file 3.** LdMNPV and OpMNPV ORFs with no homologue in LyxyMNPV genome. Numbers of LdMNPV and OpMNPV ORFs were taken from Kuzio et al. [14] and Ahrens et al. [23].

| LdMNPV | | |  | OpMNPV | | |
| --- | --- | --- | --- | --- | --- | --- |
| ORF* | Length (aa) | Name |  | ORF* | Length (aa) | Name |
| 6 | 80 |  |  | 4 | 63 |  |
| 8 | 119 |  |  | 5 | 131 |  |
| 10 | 73 |  |  | 7 | 77 |  |
| 13 | 66 |  |  | 8 | 146 |  |
| 24 | 208 |  |  | 9 | 160 | *ptp-2* |
| 31 | 59 |  |  | 10 | 220 | *ptp-1* |
| 52 | 299 |  |  | 15 | 197 |  |
| 67 | 218 | *hrf-1* |  | 28 | 56 |  |
| 73 | 90 | *bro-e* |  | 32 | 593 |  |
| 74 | 129 | *bro-f* |  | 33 | 62 |  |
| 112 | 85 | *bro-h* |  | 36 | 228 |  |
| 113 | 346 | *bro-i* |  | 37 | 141 |  |
| 114 | 403 | *bro-j* |  | 38 | 459 |  |
| 115 | 238 | *bro-k* |  | 41 | 275 | *iap-1* |
| 121 | 78 |  |  | 46 | 171 |  |
| 126 | 55 |  |  | 47 | 498 | *gta* |
| 134 | 122 |  |  | 48 | 63 |  |
| 146 | 353 | *bro-l* |  | 49 | 120 |  |
| 147 | 359 | *rr2a* |  | 51 | 81 |  |
| 148 | 596 | *rr1* |  | 52 | 109 |  |
| 149 | 53 | *ctl-1* |  | 53 | 307 | *pcna* |
| 150 | 243 | *bro-m* |  | 60 | 82 |  |
|  |  |  |  | 66 | 60 |  |
|  |  |  |  | 67 | 79 |  |
|  |  |  |  | 68 | 61 |  |
|  |  |  |  | 75 | 55 |  |
|  |  |  |  | 76 | 84 |  |
|  |  |  |  | 77 | 172 |  |
|  |  |  |  | 82 | 104 |  |
|  |  |  |  | 87 | 55 |  |
|  |  |  |  | 88 | 151 |  |
|  |  |  |  | 89 | 249 |  |
|  |  |  |  | 92 | 279 |  |
|  |  |  |  | 98 | 185 |  |
|  |  |  |  | 106 | 118 | *iap-4* |
|  |  |  |  | 110 | 94 |  |
|  |  |  |  | 113 | 361 |  |
|  |  |  |  | 114 | 424 |  |
|  |  |  |  | 117 | 97 |  |
|  |  |  |  | 118 | 202 |  |
|  |  |  |  | 120 | 82 |  |
|  |  |  |  | 121 | 70 |  |
|  |  |  |  | 122 | 243 |  |
|  |  |  |  | 123 | 211 | *lef-7* |
|  |  |  |  | 126 | 509 | *gp64* |
|  |  |  |  | 128 | 103 | *gp16* |
|  |  |  |  | 135 | 250 |  |
|  |  |  |  | 136 | 53 | *ctl-1* |
|  |  |  |  | 143 | 218 | *hrf-1* |
|  |  |  |  | 147 | 285 | *oep32* |
|  |  |  |  | 148 | 236 | *opep25* |
|  |  |  |  | 149 | 75 | *p8.9* |
|  |  |  |  | 150 | 76 |  |
|  |  |  |  | 151 | 405 | *ie-2* |
|  |  |  |  | 152 | 307 | *pe38* |
