# Supplementary figures and images for: Genomic sequencing and analyses of Lymantria xylina multiple nucleopolyhedrovirus
Source: BMC Genomics. 2010 Feb 18;11:116. doi: 10.1186/1471-2164-11-116 (PMC2830988; doi:10.1186/1471-2164-11-116)

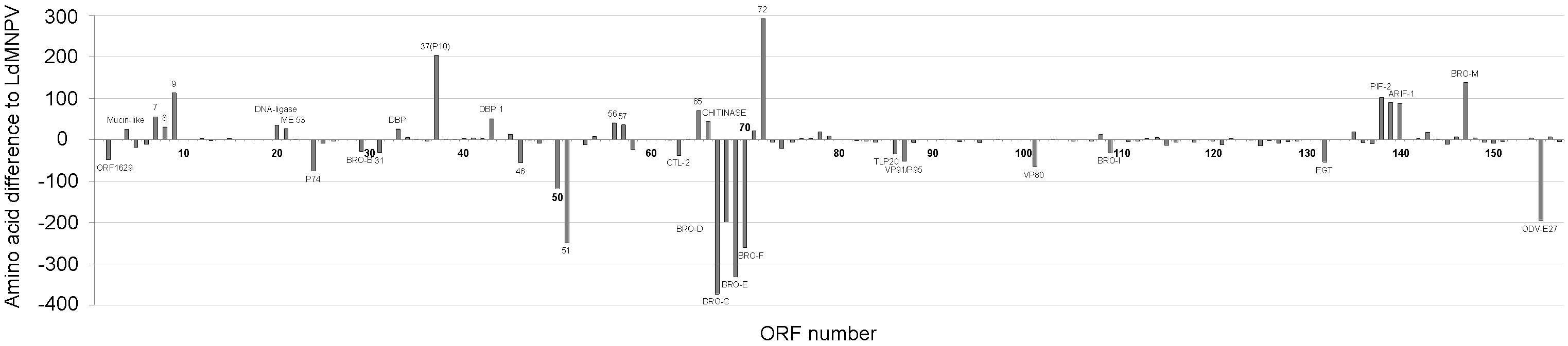

Supplement: Additional file 4 — The difference in the number of amino acids in the LyxyMNPV genome compared with the LdMNPV genome. Bars below zero show smaller ORFs in LyxyMNPV compared to LdMNPV. This file shows the difference in the number of amino acids in the LyxyMNPV genome compared with the LdMNPV genome. [file 1471-2164-11-116-S4.TIFF]
